# Impact of nephrolithiasis on kidney function

**DOI:** 10.1186/s12882-015-0126-1

**Published:** 2015-08-28

**Authors:** Vaka K. Sigurjonsdottir, Hrafnhildur L. Runolfsdottir, Olafur S. Indridason, Runolfur Palsson, Vidar O. Edvardsson

**Affiliations:** Faculty of Medicine, School of Health Sciences, University of Iceland, Reykjavik, Iceland; Children’s Medical Center, Landspitali – The National University Hospital of Iceland, Reykjavik, Iceland; Division of Nephrology, Internal Medicine Services, Landspitali – The National University Hospital of Iceland, Reykjavik, Iceland

**Keywords:** Chronic kidney disease, Diabetes, Hypertension, Nephrolithiasis, Obesity

## Abstract

**Background:**

Kidney stone disease has been associated with reduced kidney function and chronic kidney disease (CKD). The objective of the study was to examine kidney function, body mass index (BMI) and the prevalence of cardiovascular disease, hypertension and diabetes in recurrent kidney stone formers.

**Methods:**

A cross-sectional, case-control study comparing measures of kidney function, BMI and comorbid conditions was conducted in 195 kidney stone patients aged 18 to 70 years with recurrent clinical stone events and 390 age- and gender-matched controls. Wilcoxon-Mann-Whitney, chi-square tests and analysis of covariance were used to compare serum creatinine (SCr) and estimated glomerular filtration rate (eGFR) between the groups.

**Results:**

The median age of stone formers was 51 (range, 19–70) years and 108 (55 %) were males. Seventy patients (36 %) had experienced 2–4 clinical stone events, 41 (21 %) 5–10 episodes and 84 (43 %) more than 10. The median SCr was 75 (41–140) μmol/L in the stone formers and 64 (34–168) μmol/L in the control group (p < 0.001). The mean eGFR was 87 ± 20 and 104 ± 22 mL/min/1.73 m^2^ in the stone formers and controls, respectively (p < 0.001). After adjustment for body size and comorbid conditions, the difference in SCr and eGFR between cases and controls remained highly significant (p < 0.001). The prevalence of CKD was 9.3 % among stone formers compared with 1.3 % in the control group (P < 0.001). Hypertension and diabetes were significantly more prevalent among the cases that also had higher BMI than controls.

**Conclusions:**

Recurrent kidney stone formers have a significantly lower level of kidney function and a markedly higher prevalence of CKD than age- and gender-matched control subjects. The observed deleterious effect of kidney stones on kidney function appears to be independent of comorbid conditions.

## Background

Kidney stone disease is a common chronic disorder associated with painful stone episodes, lost work days and significant health care costs [1], affecting up to 10–12 % of men and 5–6 % of women [2]. In recent years, kidney stones have been associated with increased risk of chronic kidney disease (CKD) [3], and even end-stage renal disease (ESRD) [4, 5]. Furthermore, a number of studies have reported an increased prevalence of cardiovascular disease [6], diabetes, hypertension and obesity [7–9] in stone formers, suggesting that kidney stone disease may be a systemic disorder linked to the metabolic syndrome [7]. However, the independent contribution of kidney stone disease to cardiovascular risk may be difficult to detect as it may be mediated by other health conditions such as CKD in the stone-forming population [10].

Although monogenic causes of nephrolithiasis frequently result in ESRD [11], the relationship between impaired kidney function and the common type of kidney stones is much less clear. A few studies have suggested at least a two-fold risk of CKD in subjects with idiopathic calcium kidney stone disease [4, 12, 13]. Moreover, a retrospective case-control study based on data from the Rochester Epidemiology Project found stone formers to have a 51 % to 68 % increased risk for a sustained elevation in serum creatinine (SCr) and for a clinical diagnosis of CKD compared with non-stone formers [3]. Another cross-sectional study showed a reduction in measured creatinine clearance in kidney stone clinic patients compared with the general population [14]. Furthermore, in a report based on the Third National Health and Nutrition Examination Survey (NHANES III), obese stone formers had a modest decrease in estimated glomerular filtration rate (eGFR) compared with non-stone formers [15]. Finally, a recent study carried out in England and Wales evaluating predictors of CKD in a large cohort (n = 1,574,749) found female stone formers to be at an increased risk of moderate to severe CKD while no such risk was seen in males [16]. However, an accurate characterization of kidney stone patients and/or assessment of potential confounders, such as age-related decline in GFR and comorbid conditions, are lacking in the above-cited studies.

The objective of this study was to compare kidney function, body mass index (BMI) and the prevalence of cardiovascular disease, hypertension and diabetes in a well-characterized population of Icelandic patients with recurrent clinical stone events and an age- and gender-matched control group from the general population.

## Methods

### Study design and setting

This was a cross-sectional, case-control study of 195 adult patients aged 18 to 70 years with recurrent kidney stone disease. A written informed consent was obtained from all participants in the study. The study was approved by the National Bioethics Committee of Iceland (NBC 03-002-V3) and the Icelandic Data Protection Authority.

### Patient recruitment and characterization of kidney stone disease

Patients with confirmed recurrent clinical stone disease were recruited from two sources, the Kidney Stone Prevention Clinic at Landspitali – The National University Hospital of Iceland (LUH) in Reykjavik and the Icelandic Kidney Stone Registry (IKSR) which was established in 2008 in conjunction with a nationwide study on kidney stone disease [2]. Recurrent stone disease was defined as a history of a minimum of two documented clinical stone events, occurring at least 6 months apart to reduce the probability of counting the same stone event more than once. A clinical stone event was defined as either acute flank pain associated with hematuria and/or the detection of a stone by a medical imaging study, or a patient-reported stone passage. In cases where a clinical stone event was not confirmed by imaging, urinary tract infection as a cause of acute flank pain was excluded.

All patients attending the LUH Kidney Stone Prevention Clinic from December 2009 to January 2013 were eligible for participation in the study. Records of all 144 patients who attended the clinic during this period were reviewed. Of 99 patients who met the inclusion criteria, 95 agreed to participate. To increase the sample size, it was decided to also include in the study patients from the IKSR with a history of more than 2 clinical stone events and a documented stone episode after January 1, 2002. Of a total of 287 eligible IKSR cases, 124 individuals residing in the greater Reykjavik area were invited to participate. Of these, 101 accepted the invitation; 16 did not reply and 7 declined. Hence, a total of 195 patients with recurrent kidney stone disease were included in the study.

The presence of renal disorders other than kidney stone disease was excluded by self-report (interview) and thorough medical record review in all the cases, and by searching the medical information systems at LUH for all ICD-9 and ICD-10 codes suggestive of a specific kidney disease. The above strategy led to the exclusion of one patient with IgA nephropathy who developed stones in a transplanted kidney, leaving the total number of cases at 195. All study subjects were interviewed by two of the investigators and underwent a brief physical examination which included the evaluation of height, weight and blood pressure. Current medication use was recorded. To compare kidney stone patients with the general population, a control group was drawn from a cohort of 1,630 community-dwelling subjects aged 29–87 years, who participated in a cross-sectional study of bone health performed in Reykjavik in the years 2001–2003 [17]. The participants in that study underwent a thorough review of their medical history, measurement of blood pressure and body composition, and laboratory testing, including a single SCr measurement. For each kidney stone patient, two controls were selected at random from this cohort, excluding those with a history of kidney stones. Since body composition and SCr are affected by age and sex, both of which are included in eGFR calculations, cases and controls were matched for these factors. In addition, we compared the distribution of BMI in our stone-forming sample with a group of 1,271 subjects from the general Icelandic population using data from a nationwide study on nutrition conducted by the Icelandic Directorate of Health for the year 2011 (available at www.landlaeknir.is).

### Evaluation of kidney function

All available SCr levels of kidney stone cases were obtained from the electronic medical record system at LUH, yielding a total of 3,320 values. SCr measurements obtained during clinical stone episodes were excluded as such events may cause acute kidney injury. The eGFR was calculated by the MDRD study equation using the lowest available SCr value within the last year of observation or SCr measured at least 3 months after a documented clinical stone event. CKD was defined as eGFR < 60 mL/min/1.73 m^2^. Two patients who only had a single SCr value available obtained during an acute stone event were excluded from the kidney function analysis along with their corresponding controls. Only a single SCr measurement was available for each control subject. All SCr values were IDMS-standardized and measured at the clinical biochemistry laboratory at LUH.

### Stone composition

Results of stone analysis were retrieved from medical records and all available medical imaging reports were reviewed in order to differentiate radiopaque from radiolucent stones. Stones visualized on a plain film of the kidney, ureters and bladder were considered radiopaque and were classified as calcium-containing stones.

### Definition of comorbid conditions

Information on comorbid conditions was obtained through medical history reported by the stone patients and the controls, review of their medical records and/or by searching for the appropriate ICD-9 and ICD-10 diagnostic codes for both cases and controls in the electronic patient information system at LUH. Hypertension was defined as a history of hypertension diagnosed by a physician and/or treatment with antihypertensive medications. Diabetes was defined as a history of a physician diagnosed diabetes and/or treatment with antidiabetic drugs. Cardiovascular disease was defined as evidence for coronary artery disease, cerebrovascular disease or peripheral vascular disease by ICD-9 (410–414, 430–438 and 440–448) and ICD-10 (I20-I25 and I60-I79) diagnostic codes suggestive of these disorders. The presence of cardiovascular disease was further confirmed by review of individual medical records.

### Statistical analysis

Data are presented as mean ± standard deviation for normally distributed continuous variables and median (range) for continuous variables that are not normally distributed. Wilcoxon-Mann-Whitney, chi-square and Fisher’s exact tests were used to compare stone formers with controls and analysis of covariance (ANCOVA) to compare the two groups with respect to SCr and eGFR, adjusting for BMI, hypertension, diabetes and cardiovascular disease which may affect the outcome variables. The same methods were used to compare a subgroup of patients with calcium stones with their corresponding control subjects. Spearman’s correlation coefficient was used to correlate the number of clinical stone events with measures of kidney function. Statistical analysis was performed with the computer software SPSS version 11 (SPSS, Chicago, IL).

## Results

Characteristics of the 195 recurrent kidney stone formers are outlined in Table 1. The median age was 51 (range, 19–70) years and 108 (55 %) were males. Seventy patients (36 %) had experienced 2–4 clinical stone events, 41 (21 %) had suffered 5–10 events and 84 (43 %) more than 10 stone events. A total of 154 patients (79 %) had radiopaque (calcified) stones and in 20 patients the stones were radiolucent. In the remaining 21 patients the radiological characteristics could not be determined. Fifty-nine (30 %) patients had stones composed of calcium oxalate but the stone composition was unknown in more than half of the cases. Of 131 patients (67 %) who underwent extracorporeal shock wave lithotripsy, 99, 18 and 10 required 1–4, 5–10 and > 10 treatments, respectively. In addition, 2 patients required open surgery for stone removal and 8 underwent percutaneous procedures. Finally, 80 patients had endoscopic ureteral catheter insertion performed 1–4 times during stone events and 4 patients on 5–10 occasions.

**Table 1:** Clinical characteristics of kidney stone formers

Age at first stone episode, years	35 (15–67)
Number of stone episodes	
2-4, N (%)	70 (36)
5-10, N (%)	41 (21)
>10, N (%)	84 (43)
Stone type (number of patients)	
Radiopaque stones, N (%)	154 (79)
Calcium oxalate	
Idiopathic, N	55
Hemicolectomy/gastric bypass, N	5
Calcium phosphate	
Idiopathic, N	7
Primary hyperparathyroidism, N	3
Unknown, N	84
Radiolucent stones, N (%)	20 (10)
Uric acid, N	13
2,8-Dihydroxyadenine, N	2
Unknown, N	5
Not determined, N (%)	21 (11)
ESWL, N (%)	131 (67)
Insertion of ureteral catheter, N (%)	84 (43)
Stone removal surgery, N (%)	10 (5)
Percutaneous, N	8
Open, N	2

Data are presented as median (range) for continuous variables and as number (%) for categorical variables

*ESWL* extracorporeal shock wave lithotripsy

Table 2 shows the comparison of kidney function and comorbid conditions between kidney stone patients and the control group. Cases and controls were almost exclusively non-Hispanic Caucasians, which is representative of the Icelandic population, and the control subjects were carefully matched for age and sex. The median SCr was 75 (41–140) μmol/L in the stone formers and 64 (34–168) μmol/L in the control group (p < 0.001). The mean eGFR was 87 ± 20 and 104 ± 22 mL/min/1.73 m^2^ in the stone formers and controls, respectively (p < 0.001). The prevalence of CKD was 9.3 % among stone formers compared with 1.3 % in the control group (P < 0.001). All cases with CKD had stage 3 disease and no difference between men (9.4 %) and women (9.2 %) was observed (p = 0.96). The number of clinical stone events and/or surgical stone removal procedures did not correlate with either SCr or eGFR. The prevalence of hypertension was significantly higher among cases than controls (p = 0.003). The distribution of BMI among stone formers was also significantly different from the general population in the year 2011, as 34.4 % of stone formers had BMI > 30 kg/m^2^ compared with 21.0 % of individuals in the general Icelandic population and 19.5 % of the control subjects (p < 0.001).

**Table 2:** Comparison of kidney function and comorbid conditions between kidney stone formers and controls

	Stone formers (N = 195)	Control group (N = 390)	P-value
Age (years)	51 (19–70)	51 (29–71)	-
Males, N (%)	108 (55.3)	216 (55.3)	-
Serum creatinine (μmol/L)^a^	75 (41–140)	64 (34–168)	< 0.001
eGFR (mL/min/1.73 m^2^)^a^	87.0 ± 20.3	104.0 ± 22.1	< 0.001
eGFR < 60 mL/min/1.73 m^2a^, N (%)	18 (9.3)	5 (1.3)	< 0.001
BMI (kg/m^2^)	28.1 (17.0-49.1)	26.0 (17.4-45.3)	< 0.001
Systolic BP (mm Hg)	130 (86–175)	131 (90–212)	0.51
Diastolic BP (mm Hg)	78 (38–108)	81 (54–122)	0.001
Hypertension, N (%)	65 (33.3)	86 (22.1)	0.003
Diabetes, N (%)	15 (7.7)	12 (3.1)	0.02
Cardiovascular disease, N (%)	10 (5.1)	28 (7.2)	0.34

Data are presented as mean ± standard deviation for normally distributed variables, median (range) for other continuous variables and as number (%) for categorical variables

Abbreviations: *BMI* body mass index, *BP* blood pressure, *eGFR* estimated glomerular filtration rate

^a^Two cases with only a single serum creatinine value available during a stone episode and the corresponding controls were excluded

**Fig 1:**
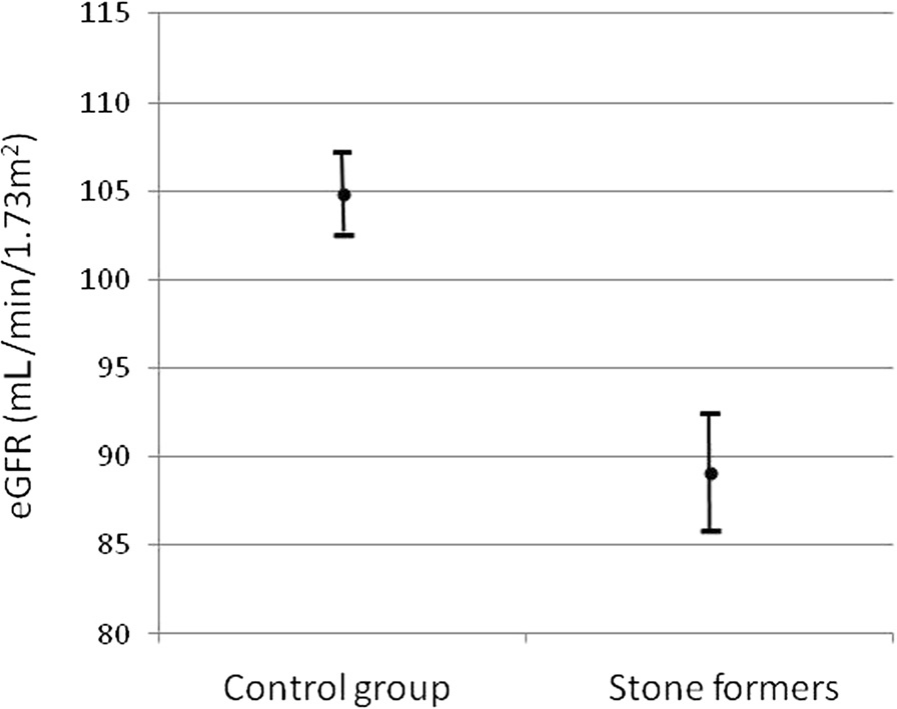
Adjusted mean eGFR in the kidney stone patients and the control group. The bars represent 95 % confidence intervals

Diabetes was more common among the cases, 7.7 % vs 3.1 % (p = 0.02), whereas the prevalence of cardiovascular disease between the two groups was similar. After adjustment for body size and comorbid conditions, the observed difference in eGFR and SCr between cases and controls remained highly statistically significant (p < 0.001, ANCOVA) as shown in Fig. 1.

A separate analysis was carried out for a subset of calcium stone formers and their respective controls (Table 3). Eight of the 154 calcium stone formers and their corresponding control subjects were excluded from the analysis because 3 had primary hyperparathyroidism as the underlying cause of nephrolithiasis and 5 had a past history of either gastric bypass surgery or hemicolectomy. For the remaining 146 cases, the median SCr was 76 (41–140) μmol/L compared with 66 (39–168) μmol/L in the control group (p < 0.001). The prevalence of CKD was higher among stone formers than controls, 5.5 % vs 1.0 % (p = 0.008). After adjustment for body size and comorbid conditions, the difference in SCr concentration and eGFR remained significant between the calcium stone formers and their controls (p < 0.001, ANCOVA). No correlation was found between the number of stone episodes and/or surgical stone removal procedures and SCr or eGFR in the calcium stone formers. The prevalence of hypertension and elevated BMI was significantly higher among calcium stone formers than controls, while the prevalence of diabetes and cardiovascular disease was not statistically different between the two groups (Table 3).

**Table 3:** Comparison of kidney function and comorbid conditions between idiopathic calcium stone formers and controls

	Calcium stone formers (N = 146)	Control group (N = 292)	P-value
Age (years)	50 (19–70)	51 (29–71)	-
Males (%)	83 (56.8)	166 (56.8)	-
Serum creatinine (μmol/L)^a^	74 (41–131)	66 (39–168)	< 0.001
eGFR (mL/min/1.73 m^2^)^a^	87.1 ± 18.9	103.8 ± 21.0	< 0.001
eGFR < 60 mL/min/1.73 m^2a^, N (%)	8 (5.5)	3 (1.0)	0.008
BMI (kg/m^2^)	27.9 (17.0-49.1)	26.0 (17.7-45.3)	< 0.001
Systolic BP (mm Hg)	129 (86–175)	130 (93–212)	0.28
Diastolic BP (mm Hg)	78 (47–108)	81 (54–122)	0.001
Hypertension, N (%)	44 (30.1)	59 (20.2)	0.02
Diabetes, N (%)	9 (6.2)	8 (2.7)	0.08
Cardiovascular disease, N (%)	7 (4.8)	19 (6.5)	0.48

Data are presented as mean ± standard deviation for normally distributed variables, median (range) for other continuous variables and as number (%) for categorical variables

Abbreviations: *BMI* body mass index, *BP* blood pressure, *eGFR* estimated glomerular filtration rate

^a^One case with only a single serum creatinine value available during a stone episode and the corresponding control were excluded

Of the 18 patients with confirmed radiolucent stones, 2 patients with adenine phosphoribosyltransferase (APRT) deficiency were excluded. Of the remaining 16 patients, 7 (44 %) had CKD compared with none of the corresponding control subjects (p < 0.001). The prevalence of CKD was also significantly higher among the patients with radiolucent stones compared with calcium stone formers (p < 0.001).

## Discussion

In the present study, we found a significantly lower level of kidney function and a markedly higher prevalence of CKD in patients with recurrent kidney stone disease than in age- and gender-matched control subjects. Nearly identical results were observed in the subset of idiopathic calcium stone formers. Moreover, kidney stone disease associated with obesity, hypertension and diabetes but not with established cardiovascular disease. The observed deleterious effect of stone disease on kidney function was independent of these comorbid conditions.

Adverse renal outcomes, ranging from modest reduction in kidney function [14] to established CKD [3] and even ESRD [4, 18], have previously been reported in patients with the common type of kidney stone disease. However, earlier studies have shown conflicting results. For example, in a large study based on data from NHANES III [15] the observed mild eGFR reduction was limited to obese stone formers, while in another study the increased CKD risk in kidney stone patients was limited to the female gender [16]. Worcester et al. [14], reported a significantly lower measured creatinine clearance adjusted for age, gender and body weight in 1856 US kidney stone formers followed at the University of Chicago compared with that of 153 normal individuals. Even though these authors did not correct for comorbid disorders, their results are in agreement with our findings. As in the current study, the average reduction in kidney function in the Chicago study was modest and the observations among calcium stone formers were similar to ours. Data on the prevalence of CKD were not reported for the Chicago cohort.

In our study, approximately 9 % of all kidney stone patients and 6 % of calcium stone formers had CKD, which is approximately 6 to 7 times higher than in the control group. These results contrast with findings of other recent studies showing stone formers to be at an approximately twofold risk of CKD [4, 12, 13] or even less [3]. We speculate that disease severity may have contributed to the increased CKD risk in our study since a large proportion of the cases had a history of both multiple stone events and stone removal procedures. Also, a low prevalence of CKD in the control group may have influenced this finding to some extent, although in these relatively young individuals the prevalence of eGFR < 60 mL/min/1.73 m^2^ is expected to be low [19]. The exclusion of all SCr measurements obtained during the first 3 months following a clinical stone event and the selection of the lowest available SCr value within the last year of available measurements for our eGFR calculations likely biased our results towards a lower CKD risk in stone formers when compared to previous work in this field, adding further strength to our findings. Interestingly, no correlation was observed between the number of stone events and/or surgical stone removal procedures and kidney function in our study. In contrast, a recent Canadian cohort study showed an augmentation of the CKD risk in patients with more than one clinical stone event compared with single stone formers [4]. However, these findings are not directly comparable to the results of our study in which all subjects had recurrent stone disease.

The degree of kidney injury associated with nephrolithiasis has been reported to vary for different stone types. We observed a significantly worse renal outcome in patients with radiolucent stones compared with other types of kidney stones. In the aforementioned study by Worcester et al., stone type was predictive of the level of kidney function which was lowest in patients with cystinuria followed by uric acid stone formers [14]. The investigators demonstrated that patients with uric acid stones had a lower measured creatinine clearance compared with brushite (calcium monohydrogen phosphate) and calcium oxalate stone formers and normal control subjects, and patients with stones composed of calcium oxalate, calcium apatite or struvite all had less kidney function than normal controls.

The pathogenesis of CKD in patients with kidney stone disease likely involves multiple different pathways [3, 20]. Obstruction to urine flow caused by stones has the potential to inflict kidney injury. In fact, unilateral ureteral obstruction, which in animal models has been shown to cause intense renal vasoconstriction and renal blood flow reduction, can lead to significant ischemia and permanent renal parenchymal damage [21]. Renal parenchymal crystal deposition and the associated inflammation and fibrosis have been well described in patients with primary hyperoxaluria and APRT deficiency [11] and similar histological changes have also been observed in patients with uric acid and struvite stones [10]. Furthermore, Evan et al. [22] have elegantly shown that obstruction of the ducts of Bellini, commonly seen in brushite stone formers, is strongly associated with tubular atrophy, interstitial fibrosis and glomerulosclerosis [23]. While crystal nephropathies can cause inflammation and fibrosis leading to severe kidney damage, the underlying pathobiological mechanisms of CKD in ordinary human kidney stone disease remain elusive. In animal models, however, the best characterized pathway of calcium oxalate crystal-induced inflammation involves NLRP3 inflammasome-mediated mechanisms, leading to direct injury to tubular cells, neutrophil recruitment, tubulointerstitial inflammation and progressive renal failure [15, 24].

In our study the prevalence of obesity, hypertension and diabetes was higher in stone formers than in the control group. The increased prevalence of these features of the metabolic syndrome in stone formers has previously been reported [3, 9, 15, 25]. While hypertension and diabetes are well known risk factors for CKD, the negative impact of stone disease on kidney function occurred independently of these comorbid conditions. These findings are in concert with those of a recent study that associated metabolic syndrome trait clustering with a greater severity of kidney stone disease and urine chemistry risk factors [7].

Strengths of the current study include the well-defined study population of recurrent stone formers and high participation rate. In addition, by using the lowest SCr in the last year of observation for each patient and excluding from the analysis those who did not have a follow-up SCr measurement available after an acute stone event, we may have avoided an overestimation of CKD prevalence in stone formers. Moreover, cases with another known underlying cause of CKD were excluded from the study sample and two age- and gender-matched subjects were used to control for age-related decline in kidney function. Furthermore, since all emergency and inpatient care in the greater Reykjavik area is centralized at a single institution, complete medical records were available for all cases and controls allowing the assessment of all major stone events and significant comorbid conditions. Finally, as the study participants were almost exclusively non-Hispanic Caucasians our results are likely to be generalizable to the white population living in Europe and North America. The main limitations of the study are a relatively small sample size and the lack of information on stone composition in a significant number of study subjects. Other limitations include potential sources of bias related to ascertainment of variables known to affect kidney function and its estimation. However, by excluding stone patients with other underlying kidney diseases and SCr values obtained during stone episodes and by using the lowest SCr available in the last year of observation for each patient, we believe we have avoided overestimation of CKD. To the contrary, the prevalence of CKD in kidney stone patients may have been underestimated. Finally, the definition of a clinical stone event as acute flank pain associated with hematuria without confirmation by imaging can be considered a limitation. However, the fact that all patients included in the study had recurrent kidney stone disease confirmed by a physician and a number of imaging studies carried out during the course of their disease makes other diagnoses unlikely.

## Conclusions

Patients with recurrent kidney stone disease experience a decline in kidney function over time and have a significantly higher prevalence of CKD than age- and gender-matched control subjects. Furthermore, the negative impact of stone disease on kidney function is greatest in patients with radiolucent stones. Although obesity, hypertension and diabetes were more frequent among patients than controls, the observed reduction in kidney function and the higher prevalence of CKD appears to be independent of these comorbid conditions. Recurrent stone formers should receive care and follow-up in a multidisciplinary kidney stone clinic. Prospective long-term outcome studies are needed to better define the association between kidney stone disease and adverse renal outcomes.

## Abbreviations

ANCOVA: Analysis of covariance
APRT: Adenine phosphoribosyltransferase deficiency
BMI: Body mass index
CKD: Chronic kidney disease
eGFR: Estimated glomerular filtration rate
ESRD: End-stage renal disease
LUH: Landspitali – The National University Hospital of Iceland
IKSR: Icelandic Kidney Stone Registry
SCr: Serum creatinine
